# The Full Impact of Rotavirus Vaccines in Africa Has Yet to Be Realized

**DOI:** 10.1093/cid/ciad017

**Published:** 2023-04-19

**Authors:** A Duncan Steele, George E Armah, Jason M Mwenda, Carl D Kirkwood

**Affiliations:** Enteric and Diarrheal Diseases, Global Health, Bill & Melinda Gates Foundation, Seattle, Washington, USA; Department of Electron Microscopy and Histopathology, Noguchi Memorial Institute for Medical Research, University of Ghana, Legon, Ghana; VPD Surveillance, World Health Organization (WHO) Regional Office for Africa (WHO/AFRO), Brazzaville, Republic of Congo; Enteric and Diarrheal Diseases, Global Health, Bill & Melinda Gates Foundation, Seattle, Washington, USA

**Keywords:** Rotavirus vaccines, Diarrhea, Africa

## Abstract

Africa bears the brunt of diarrheal mortality globally. Rotavirus vaccination rates are high across the continent and demonstrate impact on diarrheal disease reduction. Nevertheless, there is room for significant improvement in managing rotavirus vaccine coverage, in access to recognized public services such as appropriate medical care, including oral rehydration therapy and improved water and sanitation.

Rotavirus is the most significant enteric pathogen associated with severe diarrheal disease in young children, contributing over 200 000 deaths globally [1, 2]. Africa has borne the brunt with approximately 80% of the global burden of rotavirus mortality attributed to sub-Saharan Africa [3], and recent estimates record the highest rates of rotavirus-associated deaths in children in Africa (Figure 1). Notably, 8 African countries account for approximately 60% of the global mortality [3].

**Figure 1.**
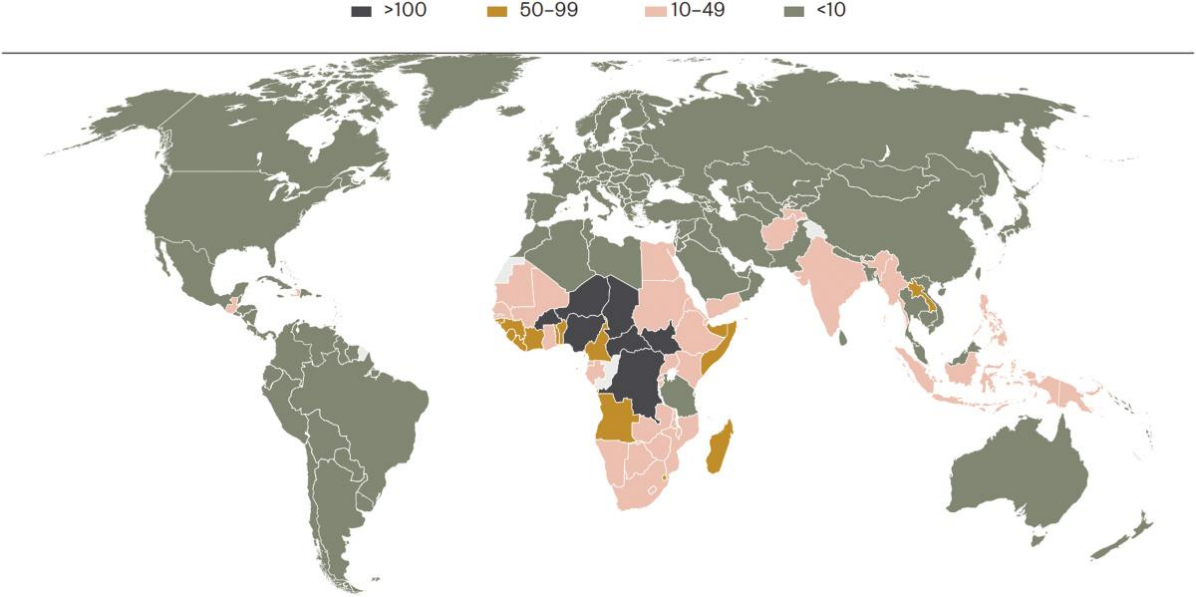
Rates of rotavirus mortality per 100 000 children under 5 in 2019, by country. Sourced from reference [3].

Fortunately, we have World Health Organization (WHO)-prequalified rotavirus vaccines that have been proven to be safe and efficacious in African populations [4–6]. African Ministries of Health, with financial support from Gavi, The Vaccine Alliance, and technical support from the WHO Regional Office for Africa (WHO/AFRO) have responded, and rotavirus vaccination is widespread in Africa with 43 of the 54 countries utilizing rotavirus vaccines in their immunization program. In August 2022, Nigeria introduced rotavirus vaccines into its national program for immunization, heralding another major milestone in the fight to reduce regional and global mortality of rotavirus diarrhea. As indicated in Figure 2, Nigeria carries much of the global burden of rotavirus-associated deaths, and the recent introduction will go a long way toward reducing total global rotavirus mortality and thus total global diarrheal mortality [3].

**Figure 2.**
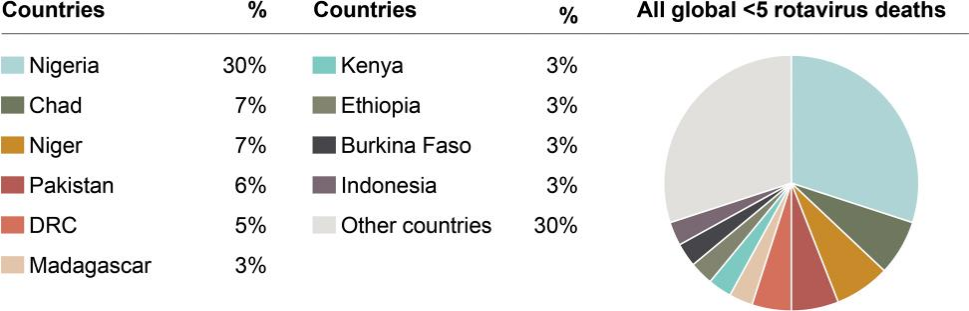
Countries with the highest number of rotavirus deaths in children under 5, as a proportion of all global deaths in 2019. Sourced from reference [3]. Abbreviation: DRC, Democratic Republic of the Congo.

Diarrheal diseases, overall, are a significant cause of mortality in young children under 5 years of age with 1 in 10 childhood deaths associated with diarrheal episodes, resulting in more than half a million childhood deaths every year [7]. Globally, the highest burden exists in sub-Saharan Africa, and reductions in diarrheal incidence over time have not been as sharp as that seen in other regions such as South Asia and Latin America over the last 2 decades [7]. Recent estimates of the global diarrheal deaths were conducted using data from the 2017–2018 WHO-coordinated Global Pediatric Diarrhea Surveillance network (GPDS) in 28 countries worldwide and incorporating estimates of country-specific diarrheal deaths from the Global Burden of Disease (GBD) study. This indicated the total number of all-cause diarrheal deaths annually for 2017 and 2018 was 582 292, and two-thirds of these (396 459 deaths) occurred in the African region [2]. Rotavirus, *Shigella*, norovirus, enterotoxigenic *Escherichia coli* and *Cryptosporidium* spp. were the top 5 attributing pathogens [2]. Several articles in this supplement highlight the ongoing importance of these pathogens in countries after rotavirus vaccination.

Well-recognized interventions to protect children from severe diarrheal disease or prevent infection remain underutilized in Africa. For instance, in a recent report with 2017 data, oral rehydration therapy (ORT) was documented as poorly applied in several countries in Africa, including countries with high diarrheal mortality [8]. Access to ORT is also variable not only across countries but also within countries in Africa. Furthermore, although access to safe water and improved sanitation facilities (WaSH) have increased globally over time between 2000 and 2017, regionally, sub-Saharan Africa showed fewer overall improvements in access. In some sub-national areas, lack of safe drinking water and improved sanitation facilities remained high [9]. Consequentially, we see a convergence of factors, including lack of real progress with access to ORT, substantial improvements in WaSH interventions, access to appropriate medical care, or improved nutrition that make African children particularly vulnerable to severe diarrheal disease and associated poor outcomes, including stunting [10].

In this issue, the Vaccine Impact on Diarrhea in Africa (VIDA) study provides further in-depth analyses in 3 African countries, Kenya, Mali, and The Gambia, with more than 10 years of surveillance and evaluation of communities through health and demographic surveillance, affirm the importance of access to safe water in the household and improved sanitation in limiting diarrheal episodes. Importantly, the study also indicates the critical role that domesticated animals and poultry play in the exposure of young children to bacterial, viral, and parasitic pathogens [11]. In addition, analysis of the case management of acute diarrheal disease and on antibiotic prescribing practices is detailed [12, 13], demonstrating suboptimal adherence to WHO guidelines for case management of acute watery diarrhea and antibiotic usage. This provides a target and motivation for national health programs to develop focused goals for improvement in infrastructure, training, and access.

Several articles in this issue highlight the critical role that numerous enteric pathogens play in causing multiple episodes of moderate-to-severe diarrhea in young African children. First, these studies included a case control design that enabled the determination of incidence rates of the specific pathogens, highlighting the importance of *Shigella*, *Cryptosporidium*, and norovirus disease in young children. Second, the studies enrolled both inpatients and outpatients attending any healthcare facility, and performed a follow up visit 60 days after enrollment [14] as well as infection leading to intestinal damage and an associated finding of stunting in children, as well as acute dehydrating diarrhea.

These in-depth case control study focused on specific communities VIDA in the 3 countries, and corroborates the data from the WHO-coordinated GPDS network, which provides estimates of the etiology-specific burden only among of hospitalized diarrhea cases. GPDS enrolled almost 8000 African children <5-year-old presenting with bloody and non-bloody diarrhea of any duration during 2017 and 2018, in 10 countries, including 4 countries in West Africa (Benin, Ghana, Cote d’Ivoire, and Nigeria), and 6 from Eastern and Southern Africa (Ethiopia, Uganda, Zambia, Zimbabwe, Mauritius, and Madagascar). Despite the introduction of rotavirus vaccines in most of these countries before 2017, rotavirus remained the leading cause of hospitalized diarrhea in seven of ten countries. However, countries that had introduced rotavirus vaccines had a lower burden of rotavirus (attributable fraction [AF] 21.3; 95% confidence interval [CI]: 18.1, 25.0) compared to countries that had not introduced the vaccine (AF 48.3; 95% CI 34.4, 65.5) [7].

One area in which Africa has excelled is in their implementation of rotavirus vaccines into national immunization programs. Countries on the continent have generated evidence of the burden of rotavirus disease for over 2 decades, highlighting the national and regional rates of infection and mortality [15], the epidemiology and early age of infection [16, 17], and the diversity of rotavirus strains circulating across the continent [18]. The technical leadership of WHO/AFRO and financial support of Gavi have driven the rapid uptake of vaccines in Africa over the last 10 years with national ministries utilizing their own data on rotavirus burden to support local decision making.

Significantly, several African countries have documented the impact of routine rotavirus vaccination on a reduction in rotavirus diarrheal deaths and hospitalizations within 2–3 years post-rotavirus vaccine introduction [19–24]. A recent report from Mozambique highlights that the implementation of a rotavirus vaccine in 2015, resulted in approximately 4600 deaths averted over 4 years and saved approximately US$3.1 M in health costs [25]. These remarkable effects were achieved with a vaccine considered to have moderate vaccine effectiveness of 52% in a population with chronic malnutrition and high human immunodeficiency virus (HIV) infection [26]. With the implementation of rotavirus vaccines in 7 of the 8 countries with highest disease burden in Africa, we should see substantial declines in rotavirus mortality over the next year or so.

Furthermore, a recent cost-effectiveness analysis of rotavirus vaccines incorporating the newly pre-qualified rotavirus vaccine products from Indian manufacturers, which are now being used in multiple African countries, demonstrated the reduction of substantial rotavirus disease burden in Africa. Rotavirus vaccines were shown to be cost-effective in all but 1 country, despite financing challenges as countries prepare for transition from Gavi support, global vaccine supply issues and the challenges associated with the pandemic [27].

Nevertheless, the VIDA studies highlight that—despite the introduction of rotavirus vaccine in all 3 countries and relatively high vaccine coverage—rotavirus was identified as a significant cause of moderate-to-severe diarrhea in infants, reproducing the early age of infection seen in Africa [16]. In addition, rotavirus was observed in the 1–2-year-old age group in significant proportions. Moreover, the ability of moderate-to-severe diarrhea to cause linear growth faltering remains unchanged post rotavirus vaccine introduction.

The modest efficacy of rotavirus vaccines in Africa are well recognized and likely due to a multitude of factors including host considerations such as malnutrition, comorbidities or environmental enteropathy [28]. The remaining fragment of severe rotavirus disease in infants and young children may well be addressed if we could improve vaccine coverage, although it was high in these studies, or consider alternative dosing schedules such as booster doses or neonatal immunization [29, 30].

An alternative approach would be to accelerate the development of next generation rotavirus vaccines, with improved efficacy in these very young ages. This is an ongoing area of research and has received a surge of excitement due to the recent successes of alternate vaccine constructs and technology platforms for the severe acute respiratory syndrome coronavirus 2 (SARS-CoV-2) vaccines. Ongoing research to evaluate next generation, parenteral rotavirus vaccine candidates should be accelerated and prioritized given the continued burden of rotavirus disease in countries that have introduced the oral, attenuated vaccines. However, optimistic timelines suggest that these new vaccines would not be available within the next 5–8 years, and so focus on improving coverage of current rotavirus vaccines to under-reached populations should be a priority.

In the meantime, new country introductions of the available pre-qualified rotavirus vaccines, such as that in Nigeria this month, and establishing high rotavirus vaccine coverage are key to the immediate short-term reductions in diarrheal deaths. Implementation of an integrated approach for diarrheal disease control to include access to ORT and zinc supplements, improved water and sanitation and improved management of diarrheal disease episodes are achievable goals, which countries can pursue with global public health support.

## Notes

***Supplement sponsorship*.** This supplement was sponsored by the Bill & Melinda Gates Foundation. This study is based on research funded in part by grants from the Bill & Melinda Gates Foundation (OPP1111236/OPP1116751). We thank the families who participated in the studies, the project field staff for their hard work and dedication, and the physicians and administration at each site who generously provided facilities for the conduct of the study.

***Financial support.*** The VIDA study is based on research funded by grants from the Bill & Melinda Gates Foundation (grant number OPP1111236/OPP1116751).
